# Factors behind job preferences of Peruvian medical, nursing and midwifery students: a qualitative study focused on rural deployment

**DOI:** 10.1186/s12960-015-0091-6

**Published:** 2015-12-02

**Authors:** Luis Huicho, Cristina Molina, Francisco Diez-Canseco, Claudia Lema, J. Jaime Miranda, Carlos A. Huayanay-Espinoza, Andrés G. Lescano

**Affiliations:** Instituto Nacional de Salud del Niño, Lima, Peru; School of Medicine, Universidad Peruana Cayetano Heredia, Lima, Peru; School of Medicine, Universidad Nacional Mayor de San Marcos, Lima, Peru; Centro de Investigación para el Desarrollo Integral y Sostenible, Universidad Peruana Cayetano Heredia, Lima, Peru; CRONICAS Centre of Excellence in Chronic Diseases, Universidad Peruana Cayetano Heredia, Lima, Peru; Project Development and Evaluation, Universidad ESAN, Lima, Peru; Salud Sin Límites Perú, Lima, Peru; School of Public Health and Administration, Universidad Peruana Cayetano Heredia, Lima, Peru; Department of Parasitology, US Naval Medical Research Unit 6 (NAMRU-6), Lima, Peru; Public Health Training Program, US Naval Medical Research Unit 6 (NAMRU-6), Lima, Peru; Batallón Libres de Trujillo 227, LI33 Lima, Peru

**Keywords:** Medical students, Nursing students, Midwifery students, Career choice, Motivations, Incentives, Rural practice, Attraction, Retention

## Abstract

**Background:**

Deployment of health workforce in rural areas is critical to reach universal health coverage. Students’ perceptions towards practice in rural areas likely influence their later choice of a rural post. We aimed at exploring perceptions of students from health professions about career choice, job expectations, motivations and potential incentives to work in a rural area.

**Methods:**

In-depth interviews and focus groups were conducted among medical, nursing and midwifery students from universities of two Peruvian cities (Ica and Ayacucho). Themes for assessment and analysis included career choice, job expectations, motivations and incentives, according to a background theory a priori built for the study purpose.

**Results:**

Preference for urban jobs was already established at this undergraduate level. Solidarity, better income expectations, professional and personal recognition, early life experience and family models influenced career choice. Students also expressed altruism, willingness to choose a rural job after graduation and potential responsiveness to incentives for practising in rural areas, which emerged more frequent from the discourse of nursing and midwifery students and from all students of rural origin. Medical students expressed expectations to work in large urban hospitals offering higher salaries. They showed higher personal, professional and family welfare expectations. Participants consistently favoured both financial and non-financial incentives.

**Conclusions:**

Nursing and midwifery students showed a higher disposition to work in rural areas than medical doctors, which was more evident in students of rural origin. Our results may be useful to improve targeting and selection of undergraduate students, to stimulate the inclination of students to choose a rural job upon graduation and to reorient school programmes towards the production of socially committed health professionals. Policymakers may also consider using our results when planning and implementing interventions to improve rural deployment of health professionals.

## Background

Peru has recently progressed to the upper middle-income country category [1] and has achieved sustained economic growth for several years, along with significant improvements in maternal and child health [2-4]. Despite this progress, rural and remote areas of the Amazon and the Andes still have high poverty rates [5] and high maternal, neonatal and under-five mortality rates [6].

Continued efforts to improve social determinants are warranted to close the remaining gaps. Additionally, the health system needs further improvement in efficiency and equity, to allow adequate implementation of public health interventions, including improved deployment of health workers in rural areas.

We showed previously that rural jobs are highly unattractive for practising doctors, nurses and midwives [7-9]. Health workers perceive rural posts as transient alternatives, with expectations to shift soon to an urban job [7]. Attraction and retention strategies should consider these perceptions if they are going to retain health workers for more than a few years, so as to contribute to measurable health impact.

Studying perceptions at the undergraduate level can inform about future expectations, motivations and preferences. They can also inform how established they are at this stage, as an additional factor that may shape later job preferences.

There is relatively scarce literature addressing incentives and motivations of students of health professions to work in rural areas, particularly in low- and middle-income countries [10-20]. Most of them focus on quantitative surveys lacking in-depth exploratory assessments [10,13,16,18,19] and address primarily medical students [13,14,19,20]. However, there is evidence that mid-level health providers perform at least as well as medical doctors in several tasks [21,22] and that the context-specific skill mix of several health cadres other than doctors may guarantee quality health care provision [23-25]. Hence, in-depth explanatory studies among several health cadres may be useful to identify relevant incentives that can further be assessed through methods such as stated job preferences [10].

We previously found that 80% of doctors practising in Ayacucho had been trained in Ica, a coastal city near Lima, the capital of Peru [7]. In contrast, about 80% of nurses and midwives practising in Ayacucho had studied in the same city [7]. Ayacucho is an Andean department with vast rural underserved areas. Based on these findings, this study aimed at identifying factors related to students’ reasons for career choice, job expectations, intrinsic motivations and incentives to work in rural areas.

## Methods

### Study design and subjects

Focus groups and in-depth interviews were conducted among medical students in their last 3 years of undergraduate training in Ica, Peru (School of Medicine, Universidad Nacional San Luis Gonzaga), and among nursing and midwifery students in their last 2 years of undergraduate training in Ayacucho, Peru (School of Nursing and School of Midwifery, Universidad Nacional San Cristóbal de Huamanga). Eligible students were identified from the official university lists, and participants were finally selected among those willing to participate.

### Setting

With 755 508 inhabitants, Ica is a predominantly coastal and urban department [26], located 325 km south of Lima. It has a human development index of 0.77, compared with 0.87 for Lima [27]. It has one public university (Universidad Nacional San Luis Gonzaga de Ica) that hosts a medical school and is the main provider of doctors for Ayacucho [7].

Ayacucho city, the capital of Ayacucho’s department, is located at about 575 km southeast of Lima. Remote mountainous and jungle areas characterize a substantive extension of the department. Out of a total of 658 400 inhabitants, about 42% of its population live in rural areas [26]. It is one of the poorest departments of the country, with a human development index of 0.59 [27]. The only public university in Ayacucho provides most of the nursing and midwifery workforce deployed in this department [7].

The health system in Peru has been described previously [7]. Briefly, it is a three-tiered system, namely the Ministry of Health, Social Security and private sector. The public sector provides health care based on semi-contributory and subsidized plans. Social security is restricted to formal employees.

Public sector health professionals are inequitably distributed, with rural and remote areas lacking sufficient numbers of health workers, particularly doctors [28]. Upon graduation, all health professionals who foresee to work in the public sector or pursue a postgraduate training have to work for 1 year in a rural post (*SERUMS*, *acronym in Spanish*) [29].

As part of the Universal Health Insurance (*Aseguramiento Universal en Salud*) strategic policy [30], new attraction and retention incentives are being considered for implementation in Peru. This also represented a timely opportunity to perform our study.

### Background theory

Our background theory is based on various assumptions described below, derived from our previous findings in Peru and other settings [7-9,31-34]. We used them to prepare the guides to focus groups and in-depth interviews. They also served as our analysis framework.

1. Students choose a health career for different reasons. They have an altruistic perception of the career, as a way to help people improve their lives through prevention or treatment of their health problems. Students also think that a health career can help them to gain professional, economic and social recognition.
2. Upon graduation, all students expect to get a job in a large urban hospital, rather than a rural job at a small health facility. Additionally, medical students have more inclination towards setting their practice in urban areas.
3. Medical, nursing and midwifery students in their early years perceive already rural jobs as unattractive. However, undergraduate students are still more inclined than health professionals to accept rural posts, provided adequate incentives are offered, because of a strong sense of solidarity towards disadvantaged populations.
4. Medical students are less willing than nursing or midwifery students to take in the future a rural job. This is related to their more frequent urban origin and to the fact they are only educated to work in clinical settings and perform clinical tasks. Health facilities in rural areas are perceived as unattractive due to the lack of professional development opportunities and the fact that clinical practice can be heavily impacted by the lack of basic infrastructure and equipment.
5. Isolated financial incentives focused on salary increases, although important, are not strong enough to persuade future health professionals to establish their professional practice in rural areas.
6. Students of rural origin or having parents with rural origin are more inclined to accept a rural job.
7. Lack of previous professional experience represents a challenge to make decisions related to job preferences. Typically, participation in such a study would require students to imagine that they are already professionals and that they are actually looking for a professional appointment.

### Sampling

Twelve focus group sessions and 20 in-depth interviews were conducted, using purposive sampling. Point of saturation was the criterion for stopping further discussions. We attempted to obtain gender and rural/urban origin balance, although a predominance of female students in the nursing and midwifery groups was anticipated [35]. Group discussions were performed first. Participants for in-depth interviews were identified from different focus group members, taking into account their degree of participation in the group discussions and their willingness to provide further input during interviews. Focus groups were assembled separately for medical students and for nursing and midwifery students.

### Tools, main themes and piloting

Guides for focus group sessions and in-depth interviews were prepared taking advantage of previous experience and on the basis of the background theory. Main themes and subthemes included are shown in Table 1. The guides were piloted with students from the same careers attending local institutions other than the target universities. After the pilots, they were rephrased and refined as needed. A questionnaire was prepared to collect basic socio-demographic information.

**Table 1:** **Topics included in the interviews and focus group sessions**

**Themes and subthemes**
A. Career choice aspects
1. Reasons underlying career choice
2. Solidarity (altruism) influence
3. Family influence
4. Role of rural origin
B. Future job expectations
1. Large and small health facilities
2. Professional development opportunities
3. Salary and other financial reasons
C. Motivations to work in a rural area
1. Solidarity feelings
2. Reciprocity principle (paying back for public investment in training)
3. Equity lens (willingness to work for poorest segments of the population)
D. Incentives to work in a rural area
1. Financial
2. Professional development
3. Family welfare
4. ICT and road infrastructure to break rural isolation
5. Infrastructure and equipment of health facility

*ICT* information and communications technology

### Fieldwork

Official lists of students were obtained at each university, and classrooms were visited with support of university authorities, academic coordinators and students’ delegates.

The field phase of the study was completed between November 2010 and March 2011. A team composed by two interviewers familiar with qualitative studies approached the students at the universities, explained the purpose of the study, selected those willing to participate and obtained informed consent. All interviews and focus groups were recorded.

### Ethical issues

The institutional ethics committee at the Instituto Nacional de Salud del Niño, in Lima, approved the protocol and the tools. Local university authorities provided official authorization, and written informed consent was obtained from all participants. Confidentiality was maintained by restricting access to participants’ identity to authorized study personnel.

### Analysis

Transcription of information obtained was performed, and a priori categories for analysis were considered for each group (*career choice aspects*, *job expectations*, *motivations to work in rural areas* and *incentives to work in rural areas*). These were the same themes included in the interviews and focus groups. Iterative discussions between the core research team, interviewers and field supervisors were held, and different interpretations and approaches were considered, according to triangulation and reflexivity principles [36]. The analysis was performed on the basis of the categories described above, by using ATLAS.ti 5.0 (Scientific Software Development GmBH, Berlin, Germany), through assemblage of a codebook. After the discussion rounds, definitive categories and subcategories were identified, reflecting the discourse of participants and the interpretation of researchers. They are shown below. The discourse of nursing and midwifery students is presented together, as they did not show outstanding differences.

## Results

Overall, we performed 10 focus group sessions with 67 participants and 20 in-depth interviews. Tables 2 and 3 show the number of participants by gender and rural/urban origin. In Ica, we completed four group discussions with 27 participants, while in Ayacucho, we performed six focus groups with 40 participants.

**Table 2:** **In-depth interview participants, by gender and by geographic area of origin**

	**Male**	**Female**	**Urban**	**Rural**	**Total**
Medical students	4	4	4	4	8
Nursing students	2	4	4	2	6
Midwifery students	2	4	4	2	6
Total	8	12	12	8	20

**Table 3:** **Focus groups participants, by gender and by geographic area of origin**

	**Male**	**Female**	**Urban**	**Rural**	**Total**
Medical students	15	12	13	14	27
Nursing students	6	16	11	11	22
Midwifery students	6	12	8	8	18
Total	27	40	32	33	67

We summarize in Table 4 the intrinsic motivations and incentives to work in a rural area by career group, in terms of relevance expressed by participants. In the next sections, we expand on the discourse that emerged through the individual and group sessions.

**Table 4:** **Intrinsic motivations and incentives to work in a rural area by career group, in terms of relevance expressed by participants**

**Incentives**	**Medical students**	**Nursing/midwifery students**
Intrinsic motivations		
Pro-social	++	++++
Philanthropic	++	++++
Financial		
Salary	+++++	+++
Allowance for housing and mobility	++	++
Non-financial		
Complete equipment	++	+++
Road infrastructure	+++	+++
Professional development opportunities	+++	++
Permanent position	+++	+++
Access to ICT	+	++
Combined		
Salary + equipment	++++	+++++
Salary + road	++++	++++
Salary + professional development	++++	+++
Salary + professional development + permanent position	+++++	+++++

*ICT* information and communications technology

### Career choice

#### Medical students

Medical students consistently expressed their early vocation for patients’ care as a reason for choosing their career. They perceived medicine as an appealing profession that can restore health.

“I have always been excited with the prospect of helping people, of curing persons, of manipulating medical tools, you know”. Female, rural origin.

They also said that becoming doctors would be a straight way to improve their economic, professional and social status.

“I knew that by studying medicine I would become a respected professional, I would improve my income, and I would be a respected person”. Male, urban origin.

Medicine also seems to attract students because they perceive it as a discipline allowing them to understand how the human organism works and as a mean to persuade people to adopt healthy life styles.

“I was intrigued by how the human body works. I realized that there is much ignorance and misunderstanding about it. This may lead to worsening of diseases. I believe that we can educate people, so they can avoid diseases and enjoy a healthy life”. Male, urban origin.

Family influence was also an important factor for career choice.

“I have an uncle, gynecologist. He has been very close to our family. He must have influenced on my decision to study medicine”. Female, rural origin.

#### Nursing and midwifery students

Expectations to improve their socioeconomic condition through their professional practice were less frequently expressed in comparison with medical students as a reason to career choice. This was usually stated along with other reasons, such as family influence and the desire to serve poor communities.

“I chose my career because I wanted to help people, just like my mother. She is also a nurse. Mhhh, maybe also as a way of securing an income”. Male, urban origin.

Family members working in the health sector might also have influenced their career choice, even since their childhood.

“Mhhh… my mother works at a health center. I used to go there with her since I was a child, and I liked what people were doing in those facilities”. Male, rural origin.

The prospect of helping to restore people’s health was also mentioned, as well as the prospect of establishing a unique communication channel with their future patients.

“There is something unique about, you know, about the communication and the relationship with patients. No one else can achieve this. This can allow curing, saving lives. And I like this”. Female, rural origin.

The desire of overcoming discrimination and hostile attitudes towards poor and vulnerable people was also expressed as a reason for choosing a nursing or midwifery career.

“… when I went to hospitals, I realized that patients may not find sympathetic and friendly health professionals, especially if they are poor, or indigenous… Something is wrong, we need to improve this, it is our duty”. Female, rural origin.

#### Career choice: similarities and differences between career categories

All groups concurred that important factors driving their career choice included their desire to help people, the influence of early life experiences and the influence of health professionals acting as role models. Vocation of service was a strong reason for career choice by nursing and midwifery students, while economic and social status expectations seemed to be more powerful factors for medical students.

### Expectations towards rural service

#### All cadres

There were mixed feelings about the prospect of taking up a rural post (SERUMS) after graduation. Participants acknowledged it as a retribution for the investment made in their training and also as an opportunity for gaining professional experience in remote areas. They also thought SERUMS was a unique opportunity for discovering the local culture and for becoming familiar with local health problems.

“All health workers must complete their SERUMS, it is the way we pay what the country spent in our training. Besides, if we don’t go to a rural area, then we can’t work [for the government], we can’t apply for postgraduate [specialty] studies”. Medical student, male, urban origin.

“I know that we have to work with poor communities, as a retribution… But at the same time, each community has its own culture, then you can learn a lot from them, you discover local health problems…”. Nursing student, female, urban origin.

SERUMS was also perceived as a transient post in rural settings full of constraints and challenges. Moreover, participants imagined that SERUMS can provide them the opportunity to make up their minds about their professional future.

“Well, SERUMS is only temporary, only for one year. It is not easy to become used to those places. They often speak Quechua and you don’t, and it’s hard to become adapted. Salary is minimal; distance to city makes travels very hard. Health posts are poor, ill-equipped, you are often alone. After a while, you have already learnt a lot and you want to get a specialty, in a large city. But while you are performing your SERUMS, you think about your future, it helps you decide what you will do afterwards…”. Medical student, male, rural origin.

### Job expectations

#### Medical students

Once graduated, medical students would prefer to work in large, urban public hospitals, which may allow them to accumulate clinical experience to develop a professional career.

“I don’t have a definitive choice yet. But I would like to be in contact with patients, and I guess that for me the best thing would be to work in an urban referral hospital, where you can see more patients”. Male, urban origin.

They also expressed their preference for large hospitals from the Social Security over those from the Ministry of Health, due to perceived greater financial incentives.

“Social Security hospitals offer more benefits to their professionals… and there, they get their regular salaries plus two additional ones. That’s better than the salaries you get at the Ministry of Health”. Female, rural origin.

#### Nursing and midwifery students

Nursing and midwifery students, compared with medical students, particularly those of rural origin, showed more willingness to work in a rural area, as an expression of solidarity.

“¿Where to work once graduated? Well, I would go back where I was born, to put into practice what I have learnt, to help solving people’s problems, at least in part”. Male, rural origin.

### Motivations to work in a rural area

We considered as motivations those factors “intrinsic” to students, not immediately explained by external factors related to some way of retribution that would contribute to personal, professional and social development. Intrinsic motivations have also been defined as “the desire to do something for its own sake” [11] or as pro-social and philanthropic motivations [37].

#### Medical students

They felt that better health can contribute to improve life conditions of people. This motivation was more explicit and consistent in those of rural origin or with parents of rural origin.

“I always had in mind that once graduated I should go to a rural community, because by preventing and curing diseases, I could help people to improve their lives”. Female, rural origin.

“…Once you finish your career, you are willing to help, you can help most where people is poorer, with lower education, with few comfort. Helping them to be healthier will contribute to poverty improvement”. Female, rural origin.

#### Nursing and midwifery students

Unlike medical students, they felt that they could contribute to the health of the community, though not necessarily to improve their socioeconomic conditions.

“In a rural area, what we could do is offer people health advice, health care. We are not going to reduce their poverty, but at least we can help them with health promotion and prevention, we can treat their health problems”. Female, rural origin.

### Incentives to work in a rural area

#### Medical students

An adequate salary was considered the main incentive to work in a rural area, along with other non-financial incentives, such as factors facilitating the accomplishment of their professional duties. The achievement of acceptable personal and family life standards was also expressed as an important incentive.

“As a doctor who studied for several years, my main incentive would be a good salary, along with adequate working conditions to perform my clinical duties. I would also expect to receive incentives leading to acceptable life conditions for myself and my family”. Male, urban origin.

Improvement of geographic accessibility and of road infrastructure was considered important to decrease the disadvantages of living in an isolated rural area and also to facilitate referral of patients.

“I would go to a rural area if there is a road that makes easier and safe to go to the city. I mean good roads, not the bad ones there are in those areas. Second, a health facility with adequate infrastructure and with complete equipment, so we can really help patients, so we can stabilize those with severe problems, and then refer them safely, quickly, in an ambulance, right?” Female, rural origin.

Getting a permanent urban job and bonus points when applying to a specialty programme after a work period in a rural area were perceived as important incentives. However, medical students expressed their reluctance to wait for too long before being promoted to a permanent position.

“I would expect to get a permanent job, but preferably in an urban hospital, because after 2 or 3 years in a rural health facility, I think I would deserve to move to a better place. I would also expect to get some incentives for applying to a specialty”. Male, urban origin.

#### Nursing and midwifery students

Their salary expectations were lower than those of medical students, and the importance of concurrent non-financial incentives was also expressed. They put more emphasis on equipment and infrastructure, which would allow them to achieve better clinical performance. The relevance of access to information technology and to professional training opportunities was also highlighted.

“Well, you need to get an income for satisfying your daily needs: food, travel, accommodation. You also need saving for the sake of yourself and your family. We are not asking for the amount a doctor would receive, but we would expect a decent salary at least. I would also expect a decent health facility, with all equipment for performing my duties”. Male, rural origin.

“Salary, yes, absolutely. But also periodic opportunities for accessing postgraduate courses and online training, to ensure that you are not lagging behind, that you keep having a good clinical performance”. Male, rural origin.

## Discussion

### Career choice

#### Medical students

It is encouraging that a genuine concern for others is still a prominent reason for choosing a medical career, an asset that should be reinforced during undergraduate training through frequent contact with underserved communities, as it is occurring in some medical schools in Peru [38]. This is important to avoid the decline of idealism in students, which unfortunately seems to occur since the early years of medical training [39], affecting specifically the interest of students to work for underserved communities, their sense of responsibility for the health of society as a whole and their perception of the medical profession as a powerful tool for social service.

Early life experiences may influence a career choice in medicine and other fields [40,41]. This is an insufficiently explored area and deserves consideration as part of training efforts of future generations of health professionals. The role of family factors in the decision to choose a medical career, previously reported [31,40,42], has also been found in our study, reflecting the positive role that family models play in shaping young people’s career choice decisions.

Our medical students believed that by becoming doctors they can guarantee their later professional, economic and social status, which is at odds with the generalized perception that medicine does not guarantee anymore a profitable future [31,42,43], unless doctors resort to multiple practice appointments [42,43]. This may be due to the fact that students have not experienced yet the actual constraints imposed by the labour market, which privileges professions related to productive and technological activities [43]. It may also be related to the fact that our participants lived in cities with lower economic development and living standards compared with the national levels, which would explain at least in part their lower income expectations.

The academic appeal of medicine has been reported in other settings [44,45], and according to our study, it remains an influence on career choice. This positive perception of medical profession should be complemented by medical schools during undergraduate training through strong messages emphasizing the social role of medicine.

#### Nursing and midwifery students

Nursing and midwifery students are aware that their future salary standards and scope of practice are more limited than those of doctors. They also show higher social service motivations. These findings are in line with other studies showing that nursing and midwifery career choices seem more related to factors other than exclusive financial gains [46-48]. Academic institutions should take advantage of this higher idealism and social service motivation to foster the willingness to work in rural settings. Therefore, nursing and midwifery students seem more influenced by reasons related to the desire to help, to establish sympathetic relationships with patients and to relieve in some way what they perceive as social inequalities in the provision of health care [48,49].

The influence of early life experiences and role models also represent an opportunity window for fostering early positive perceptions towards nursing and midwifery careers.

### Expectations towards rural service

#### All cadres

While medical students showed preference for working in large urban hospitals, they did not express simultaneous interest in private practice or in dual practice, in contrast with what has been reported in other studies [50], probably because they are not part of the labour market yet. Dual practice is widespread in Peruvian urban settings and needs serious consideration when discussing health workforce reforms, including better regulation of professional practice [51].

Academic institutions should consider changes in undergraduate curriculum to reinforce rural job expectations. Likewise, policymakers should not overlook the strong expectations towards urban positions expressed by all students, and they should consider including as a potential incentive the offer of an urban post after a given period of rural practice. Otherwise, the probability of rural retention would be jeopardized by the expectation of young doctors in pursuing a specialist training instead of choosing a rural job, which has been reported recently in Peru [52] and was confirmed by our study.

Rural service SERUMS is a 1-year appointment, a period perceived as insufficient to make a real difference for people’s health. The Peruvian government has been considering plans to extend the SERUMS duration to 3 years. However, effective steps to counteract the reluctance of health professionals to remain in remote and rural areas with inadequate health facilities and life conditions need serious consideration and implementation [53]. This includes systematic efforts by academic institutions to reinforce students’ altruism and solidarity, through reinforcement of rural rotations, and through a stronger presence of global health, medical humanities, sociology, anthropology and ethics in the medical curriculum, to ensure that bold messages on equitable health care as a basic human right are delivered.

### Job expectations

#### Medical students

In Peru, health professionals working at the Ministry of Health earn substantially lower wages than those serving in the Social Security, a fact that leads to a substantial health workforce migration from the former to the latter sector [54]. This salary gap should be addressed when planning attraction and retention strategies in developing settings with three-tiered health systems [55-57].

#### Nursing and midwifery students

The intrinsic solidarity showed in our study by nursing and midwifery students was also reported elsewhere [11]. This characteristic, along with the prospect of making clinical decisions in rural jobs when there is no doctor, should be considered when designing attraction and retention strategies. Rural deployment and retention efforts should pay special attention to nurses and midwives, considered key members of the essential health teams in Peru, as it has shown that they are able to provide quality of health care [21], a fact that is particularly important in rural areas with chronic scarcity or absence of doctors. Importantly, health professionals of rural origin should be particularly targeted for rural enrolment when implementing retention interventions.

### Motivations to work in a rural area

Intrinsic motivations have been described previously in medical, nursing and midwifery students [17,18,58,59]. However, they have been somewhat neglected when planning attraction and retention strategies for different health professions [11,37,59]. Intrinsically motivated health workers may be more amenable to achieve better clinical performance, provided they are adequately trained, receive supportive supervision and receive adequate extrinsic incentives [60]. Such motivations are particularly stronger in nursing and midwifery students, who therefore should be targeted with greater emphasis by rural deployment interventions.

Resorting to altruism has recently been proposed as an alternative strategy to improve undergraduate enrolment and permanence in the career, particularly for nurses in developing countries [61]. Moreover, particular efforts should be made to enrol students of rural origin in the entry selection process, and their greater disposition to serve rural people should be reinforced during the training process. Academic institutions and policymakers should seriously consider this factor when planning attraction and retention strategies.

### Incentives to work in a rural area

#### Medical students

Our study confirmed that combined rather than isolated incentives are stronger to persuade our future doctors to choose a rural job, which is in agreement with previous reports [7-10,33,62]. We identified various non-financial incentives that could be combined with financial incentives, such as health facility equipment, family welfare, professional development opportunities, access to information and communications technology, improved road infrastructure and access to a permanent job, among others. Students also feel that after a period of service in a rural area, they deserve to be transferred to an urban post and to get preferential incentives. These findings provide a clear message to policymakers: attraction and retention strategies must be planned and implemented considering bundled incentives, which may vary from setting to setting and even within poor areas.

#### Nursing and midwifery students

We also found that in this group combinations of adequate salaries with a wide range of other incentives are more powerful than isolated incentives, which confirmed similar results showed previously in graduate nurses and midwives [7-9]. Interestingly, our nursing and midwifery students insisted in the provision of adequate equipment at health facilities as an additional attractive, likely related to their strong intrinsic motivations.

### Lessons and opportunities

We are aware that a qualitative study like this one has limitations to discriminate accurately the relative strength of different incentives, while alternative approaches such as discrete choice experiments focused on stated job preferences can identify them. We expect to report separately such preferences by combining the information found in our qualitative results with the published evidence and with current national and subnational rural deployment and retention policy initiatives.

We also acknowledge that our comparison of medical students and nursery and midwifery students may be affected by the fact that our participants are from two different regions, with different socioeconomic and cultural characteristics. While Ica is comparatively richer and more urban than Ayacucho, rural areas represent still a substantial proportion of this latter department. Therefore, our results should be interpreted within the framework of these contrasting contexts.

Both common and singular discourses found in medical, nursing and midwifery students should be considered by academic institutions when planning their enrolment and training programmes and by policymakers when planning implementation of attraction and retention strategies. The current emphasis of undergraduate training on a highly specialized and clinically oriented medicine needs to be counterbalanced through an increased promotion of a primary health care approach, with heavier focus on prevention and social aspects. Failing to take them into account can jeopardize the success chances of the current universal health coverage initiative launched by the government [30], which aims to close the equity gap of deployment that affects the rural areas.

## Conclusions

We showed a high disposition of undergraduate students to work in rural areas, which was more evident in nursing and midwifery students and in those of rural origin. Our findings may be useful to improve targeting and selection of students at educational institutions, to reshape the curriculum of these professions in aspects related to rural health with the aim of producing socially committed graduates and specifically to strengthen the disposition of students to consider a rural job once they start their professional practice. Policymakers planning the implementation of interventions to improve the rural deployment of health professionals can also benefit from our results.
